# Exposure to ALS-FTD-CSF generates TDP-43 aggregates in glioblastoma cells through exosomes and TNTs-like structure

**DOI:** 10.18632/oncotarget.4680

**Published:** 2015-06-28

**Authors:** Xuebing Ding, Mingming Ma, Junfang Teng, Robert K.F. Teng, Shuang Zhou, Jingzheng Yin, Ekokobe Fonkem, Jason H. Huang, Erxi Wu, Xuejing Wang

**Affiliations:** ^1^ Department of Neurology, The First affiliated Hospital of Zhengzhou University, Zhengzhou, Henan, China; ^2^ Department of Neurology, People's Hospital of Zhengzhou University, Zhengzhou, Henan, China; ^3^ Department of Pharmaceutical Sciences, North Dakota State University, Fargo, ND, USA; ^4^ College of Engineering, California State University, Los Angeles, CA, USA; ^5^ Scott & White Neuroscience Institute, Texas A & M Health Science Center, College of Medicine, Temple, TX, USA

**Keywords:** TDP-43, ALS, FTD, exosomes, tunneling nanotubes

## Abstract

Amyotrophic lateral sclerosis (ALS) and frontotemporal dementia (FTD) represent a continuum of devastating neurodegenerative diseases, characterized by transactive response DNA-binding protein of 43 kDa (TDP-43) aggregates accumulation throughout the nervous system. Despite rapidly emerging evidence suggesting the hypothesis of ‘prion-like propagation’ of TDP-43 positive inclusion in the regional spread of ALS symptoms, whether and how TDP-43 aggregates spread between cells is not clear. Herein, we established a cerebrospinal fluid (CSF)-cultured cell model to dissect mechanisms governing TDP-43 aggregates formation and propagation. Remarkably, intracellular TDP-43 mislocalization and aggregates were induced in the human glioma U251 cells following exposure to ALS-FTD-CSF but not ALS-CSF and normal control (NC) -CSF for 21 days. The exosomes derived from ALS-FTD-CSF were enriched in TDP-43 C-terminal fragments (CTFs). Incubation of ALS-FTD-CSF induced the increase of mislocated TDP-43 positive exosomes in U251 cells. We further demonstrated that exposure to ALS-FTD-CSF induced the generations of tunneling nanotubes (TNTs)-like structure and exosomes at different stages, which mediated the propagation of TDP-43 aggregates in the cultured U251 cells. Moreover, immunoblotting analyses revealed that abnormal activations of apoptosis and autophagy were induced in U251 cells, following incubation of ALS-CSF and ALS-FTD-CSF. Taken together, our data provide direct evidence that ALS-FTD-CSF has prion-like transmissible properties. TNTs-like structure and exosomes supply the routes for the transfer of TDP-43 aggregates, and selective inhibition of their over-generations may interrupt the progression of TDP-43 proteinopathy.

## INTRODUCTION

Amyotrophic lateral sclerosis (ALS) is the most common adult-onset motor neuron disease, in which loss of motor neurons leads to progressive weakness of the voluntary muscles. Frontotemporal dementia (FTD) is a form of dementia clinically characterized by behavioral dysfunction and changes in personal and social conduct. Pathological studies of ALS and FTD reveal significant neuropathological overlap, suggesting that they represent different manifestations among a spectrum of phenotypes of the similar underlying pathogenesis [1-4]. The formation of cytoplasmic inclusions which compose of misfolded proteins in neuronal and glial cells, is a key neuropathological feature of neurodegenerative diseases [5]. In the central nervous system (CNS) of patients with ALS and/or FTD, transactive response DNA-binding protein of 43 kDa (TDP-43) has been identified as a major component of the cytoplasmic inclusions [6]. TDP-43 is normally localized primarily in the nucleus, but under pathological conditions in ALS and/or FTD, TDP-43 is eliminated from nucleus and mislocated to the cytoplasm [7]. In addition, TDP-43 is also found to be modified, including cleavage, ubiquitination, and phosphorylation; ultimately this leads to its misfolding and aggregation [8]. These findings have generated new insights into the pathogenesis of a spectrum of diseases called TDP-43 proteinopathies, including Perry syndrome, inclusion body myopathy, and Paget disease of the bone. TDP-43 proteinopathies likely contribute to neurodegeneration very broadly [7]. It is well-known that TDP-43 aggregates are involved in pathogenesis of ALS and FTD; however, its formation and propagation in CNS remains largely unclear and warrants further investigation.

A breakthrough in understanding sporadic neurodegenerative disease progression was the discovery of ‘prion-like propagation’ of protein aggregates a couple of years ago [9], in which misfolded protein aggregates are excreted into the extracellular space, and then taken into neighboring cells. This cell-to-cell propagation of misfolded aggregates in CNS leads to spread of neuropathological lesions and clinical manifestations [9]. The intercellular transfer of aggregates made of tau, α-synuclein, and huntingtin (Htt) has been demonstrated; evidence for such a prion-like propagation mechanism has now spread to TDP-43 aggregates implicated in ALS and/or FTD. A recent report suggests that insoluble TDP-43 aggregates extracted from brains of ALS and FTD patients can function as seeds for cell-to-cell transmission in cells overexpressing TDP-43 [10]. Nevertheless, much is still beyond our understanding. First of all, there is currently no evidence that TDP-43 aggregates can spread between individuals to cause acquired disease in human and experimental animals. Secondly, it is still unclear about the potential mechanism underlying the intercellular transfer of TDP-43 aggregates from cell to cell.

The present study aimed to substantiate the hypothesis of prion-like propagation of TDP-43 aggregates, and further to dissect the potential mechanism underlying the intercellular transfer. We find that ALS-FTD-CSF incubation with U251 cells generates TDP-43 mislocalization, and the cell-to-cell transmission of TDP-43 aggregates is mediated via exosomes and TNTs-like structure. Furthermore, incubation of ALS-CSF and ALS-FTD-CSF with U251 causes toxic to the cells.

## RESULTS

### Intracellular TDP-43 aggregates are generated following incubation of ALS-FTD-CSF with U251 cells

**Figure 1:**
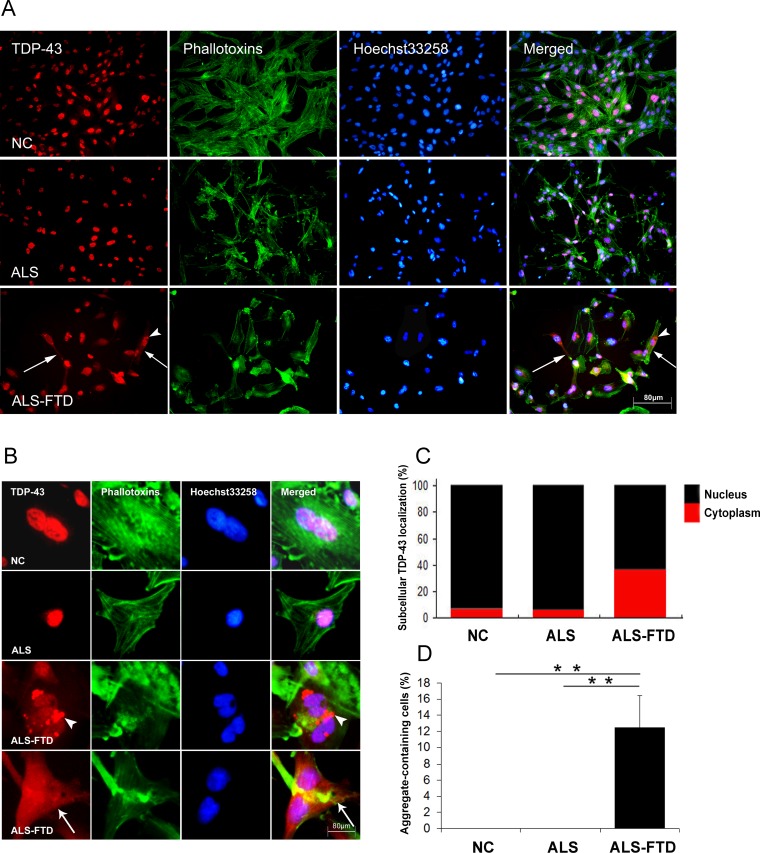
ALS-FTD-CSF induces intracellular mislocalization and aggregation of endogenous TDP-43 in U251 cells (A) Subcellular redistribution of TDP-43 in U251 cells following incubation of CSF. Immunofluorescent staining for endogenous TDP-43 labeled with rabbit anti-TDP-43 antibody (red), and the F-actin cytoskeleton labeled with phalloidin-Alexa Fluor 488 (green) were examined by fluorescent microscopy. The nucleus was stained with Hoechst 33258 (blue). Both mislocalization of TDP-43 (arrow) and TDP-43 aggregates (arrowhead) were assembled in ALS-FTD-CSF-cultured U251 cells, but the changes were not exhibited in ALS-CSF-cultured cells. Representative images of a field (n=8) are shown for one of three independent experiments from each culture. (B) High magnification microphotographs of TDP-43 mislocalization in cells following incubation of ALS-FTD-CSF. The cytoplasmic TDP-43 inclusions formed in cells and TDP-43 was diffusely distributed from the nucleus to the cytoplasm in the ALS-FTD-CSF-cultured cells. An arrowhead indicates the TDP-43 aggregates and an arrow indicates the TDP-43 distributes in the whole cells. (C) The ratio of the intensity for the fluorescence of TDP-43 located in nucleus (black) to TDP-43 in cytoplasm (red) in the CSF-cultured cells. Each bar represents values averaged from 200 cells, using student's t test with Bonferroni correction. (D) The percentage of cells containing TDP-43 aggregates following cultured with CSF. Values shown are the mean ± SD from three experiments. Level of statistical significance: **p < 0.01.

In the past 10 years, an increasing list of neurodegenerative diseases have been shown to manifest self-perpetuating seeded aggregation and spreading phenomena, the ‘prion-like’ pathological process. But there is no evidence to date that misfolded protein aggregates can spread between individual organisms to cause an acquired disease. To test whether TDP-43 aggregates can propagate via body fluid of patients, we established a CSF cell culture model using U251 cells. U251 cells were incubated for 0, 1, 3, 5, 7, 10, 14, 18, 21, and 28 day in DMEM containing 30% v/v of CSF. After exposure to CSF for 21 days, U251 cells showed a reduced rate of growth and diverse morphological changes in ALS-CSF and ALS-FTD-CSF treated groups compared with the controls (Figure 1A). We next examined the actin cytoskeleton change using phalloidin staining. Actin filaments depolymerized and labeled with fluorescent phalloidin appeared sparser and shorter as well as disorganized in ALS-CSF and ALS-FTD-CSF groups compared with NC-CSF group (Figure 1A). Also a clear disruption of the actin cytoskeleton into fragments scattered throughout the cytoplasm was observed in both ALS-CSF and ALS–FTD-CSF groups, but not in NC-CSF group (Figure 1A).

To further test whether treatment of ALS-CSF and ALS-FTD-CSF can induce the seeding of TDP-43 proteinopathy in cultured U251 cells, we performed immunocytochemistry staining for TDP-43 positive aggregates. When cells were incubated for 21 days, surprisingly, the formation of the intracellular TDP-43 aggregates and the mislocalization of TDP-43 from the nucleus to the cytoplasm were observed in the U251 cells of ALS-FTD-CSF group, but not in the ALS-CSF and NC-CSF groups, as shown in Figure 1A and 1B. Statistical results indicate that after incubation of CSF for 21 days, nearly 40 percent of TDP-43 located in cytoplasm in the cells of ALS-FTD-CSF group, while approximately 4 percent of TDP-43 located in cytoplasm in the cells of NC-CSF and ALS-CSF groups (Figure 1C). In addition, we observed more than 12 percent of cells containing TDP-43 aggregates in ALS-FTD-CSF group as shown in Figure 1D. Collectively, these results suggest that CSF from ALS-FTD patients contains the ‘seed’ for generating of TDP-43 aggregates in CSF-cultured cells. To our knowledge, this is the first evidence that protein aggregates propagate through CSF from patients with neurodegenerative diseases.

### CSF exosomal fractions from ALS-FTD patients are enriched in full-length TDP-43 and TDP-43 CTFs

**Figure 2:**
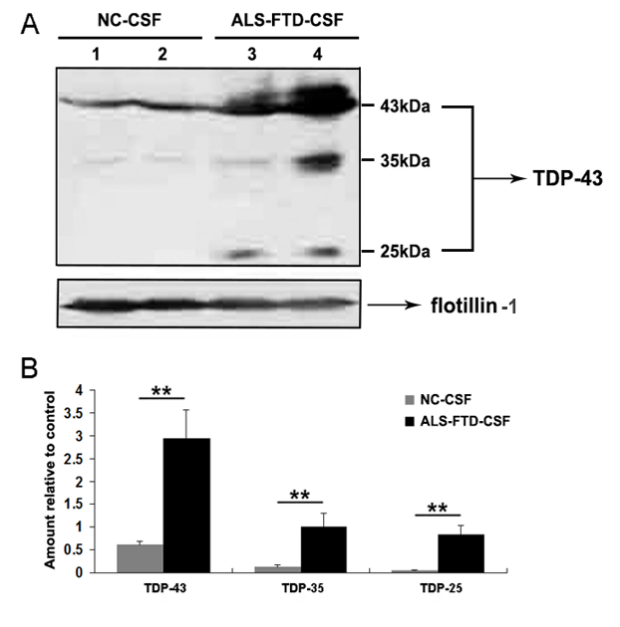
Full-length TDP-43 and TDP-43 CTFs are expressed in the exosomes derived from CSF (A) Immunoblotting analysis of exosomal fractions isolated from NC-CSF and ALS-FTD-CSF. Flotillin-1 was used as internal standard. (B) Quantification showing the significant increase of full-length TDP-43 and TDP-43 CTFs in exosomes from ALS-FTD-CSF in comparison to NC-CSF. Values shown are the mean ± SD from three experiments. Level of statistical significance: **p < 0.01.

Release of exosomes, small vesicles of endocytic origin, from a variety of different neuronal cell lines has been described [15], and the presence of exosomes in human CSF has been confirmed [14]. One of the important functions of exosomal release is the secretion of proteins, and transfer of pathogens among cells [16]. We hypothesized that exosomes derived from ALS-FTD-CSF contain the ‘seed’ propagated to the cultured cells. To test this hypothesis, we isolated exosomes from CSF by ultracentrifugation to detect the proteins associated with TDP-43 proteinopathy. Then we performed the Western blotting to detect the expression of TDP-43 and its CTFs in the exosomes. The result indicates that there was a higher enrichment of full-length TDP-43 and TDP-43 CTFs in ALS-FTD-CSF exosomes than in CN-CSF exosomes (Figure 2A, 2B). It is well known that TDP-43 CTFs lead to the formation of cytoplasmic inclusions within cells, and characteristic CTFs of insoluble TDP-43 in each disease were reproduced in a self-templating manner in cultured cells [17-19]. Collectively, we deduce that aberrant cleavage of TDP-43 in the exosomes derived from ALS-FTD-CSF may act as ‘seed’ inducing the formation of TDP-43 aggregates in the ALS-FTD-CSF-cultured cells.

### Exosomes and TNT-like structures mediate intercellular transfer of TDP-43 aggregates in ALS-FTD-CSF-cultured U251 cells

From others [20, 21], the spread mechanism of ALS and FTD lesions, explained by propagation, is classified as remote (non-contiguous) or local (contiguous) type. It is possible that the non-contiguous type propagation of toxic molecules is mediated via exosomes in blood and CSF [9]. As expected, our above mentioned results can partially explain the surmise; however, it is not clear what structure mediates cell to cell ‘domino like’ propagation of protein aggregates between neighboring cells. TNTs, F-actin-containing membranous channels, have been reported to play an important role in the intercellular spread of prions [22]. TNTs are believed to be the predominant route for the transfer of organelles and proteins between neighboring cells. Although exosomes play a pivotal role in cell-to-cell communication in both non-contiguous and contiguous types, we hypothesize that TNTs would be more efficient general machinery for communicating between contiguous cells without the need of release and uptake process.

**Figure 3:**
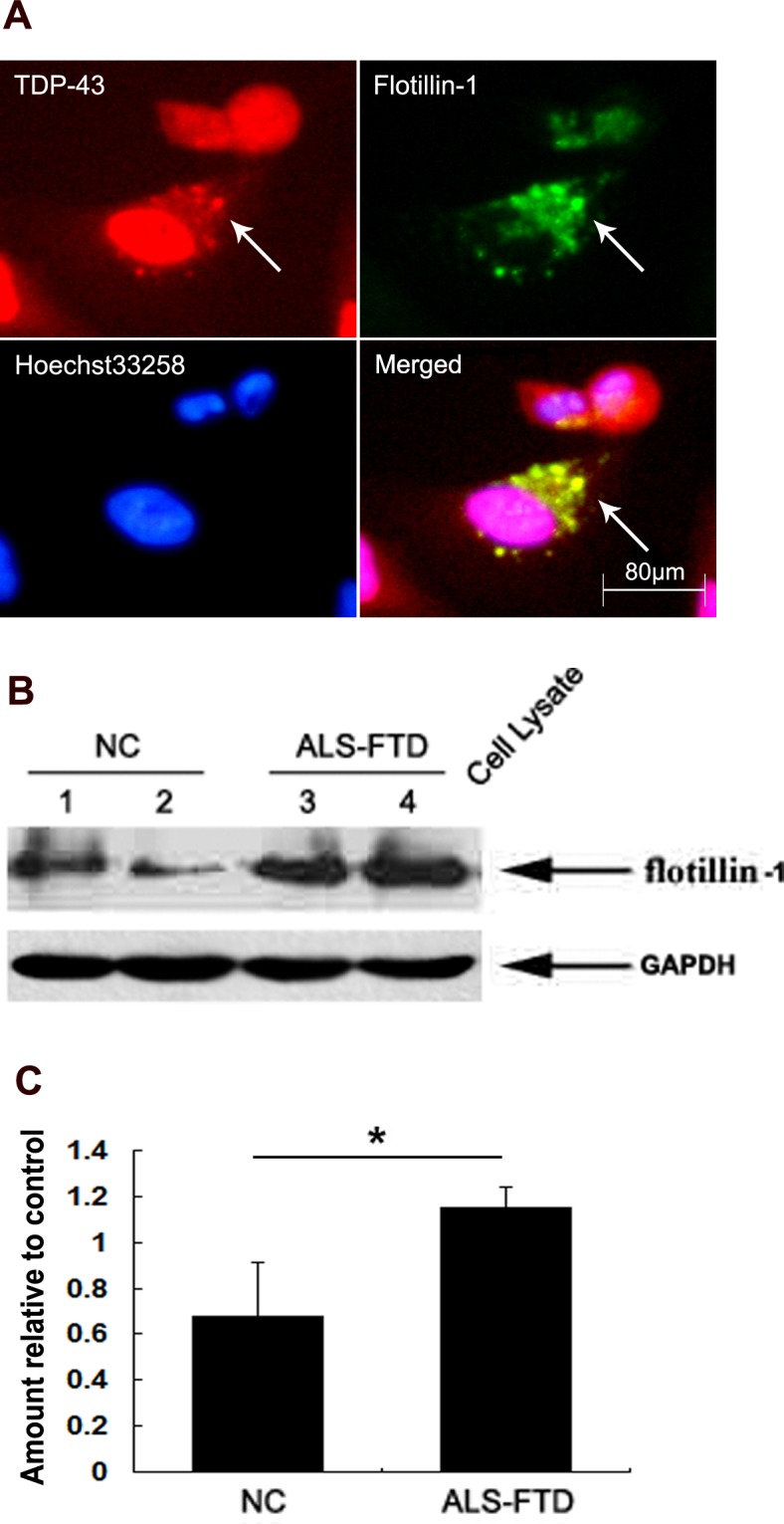
TDP-43 aggregates are propagated through exosomes (A) Localization of flotillin-1 with mislocated TDP-43 observed by fluorescence microscopy in the U251 cells following ALS-FTD-CSF incubation. Double immunofluorescence staining images shown are one representative experiment of five independent experiments performed. (B) Immunoblotting analysis of flotillin-1 expression in CSF-cultured U251 cells. The upper panel shows the increased expression of flotillin-1 in the cells exposed to ALS-FTD-CSF compared to the control groups. The lower panel shows GAPDH band used as a loading control. (C) Quantification showing significant increase of flotillin-1 in ALS-FTD-CSF group in comparison to NC-CSF. Values shown are the mean ± SD from three experiments. Level of statistical significance: *p < 0.05.

**Figure 4:**
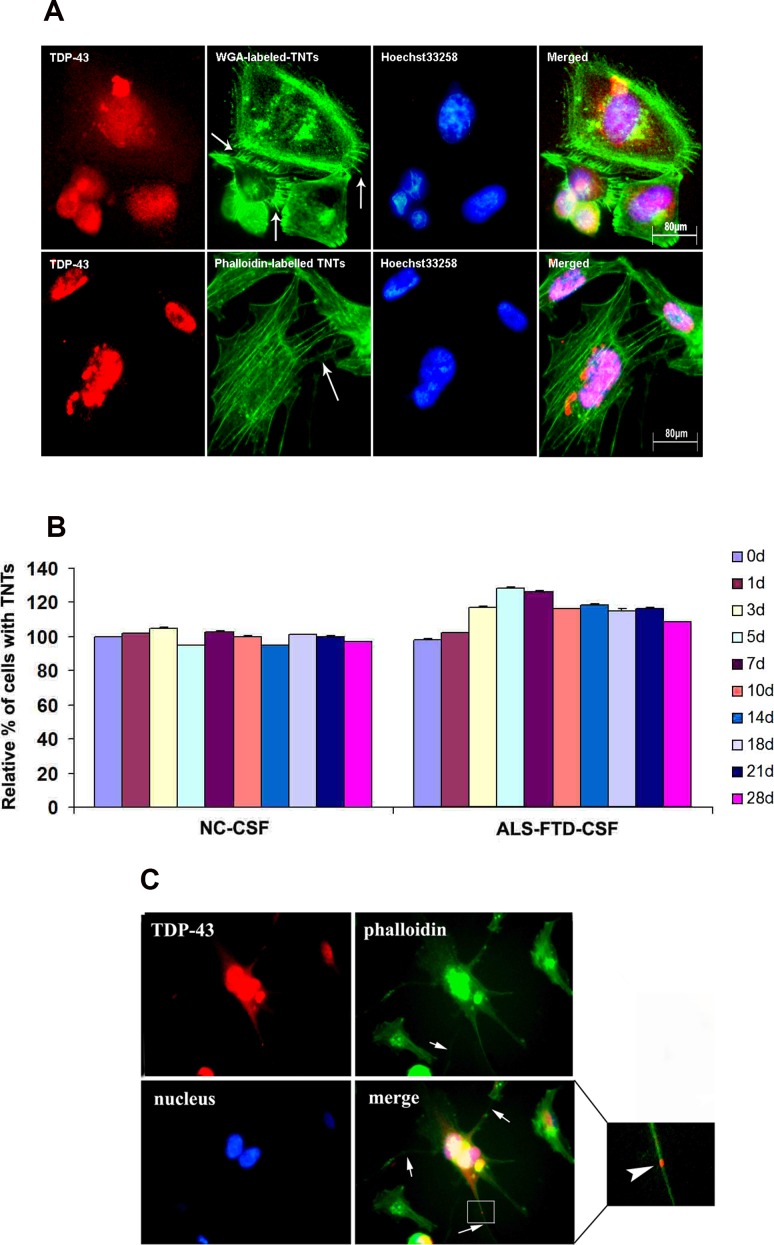
TDP-43 aggregates are transmitted through TNTs-like structure (A) Alexa Fluor 488 conjugated-WGA (top image) and Alexa Fluor 488 conjugated–phalloidin (bottom image) stained TNTs-like structures were analyzed by fluorescence microscope following ALS-FTD-CSF incubation of U251 cells. Fixed cells are connected to surrounding cells by numerous ultrafine membrane extensions, namely TNTs-like structure (arrows). (B) Percentage of TNT-connected U251 cells at various time points during exposure of NC-CSF or ALS-FTD-CSF. Values shown are the mean ± SD from three experiments. (C) Transfer of TDP-43 aggregates occurs through TNTs-like structure in ALS-FTD-CSF-cultured U251 cells. Cells were stained with Alexa Fluor 488 conjugated–phalloidin (green) to label TNTs-like structure and rabbit polyclonal antibody (red) to label TDP-43 aggregates. TDP-43 aggregates were found inside TNTs-like structure adjacent cells. The amplified-field image is shown in the right panel. The staining merge indicates that TDP-43 aggregates are present within the lumen of the TNTs-like structure (enlarged views of the boxed areas, aggregates are clearly present within the lumen of the TNTs-like structure).

To test the hypothesis, we first investigated whether flotillin-1, a lipid raft marker, is increased in U251 cells following incubation with ALS-FTD-CSF. Immunoblotting analysis showed the expression level of flotillin-1 significantly higher in the cells following ALS-FTD-CSF incubation than NC-CSF for two weeks (Figure 3B, 3C). Next we evaluated whether exosomes mediated the TDP-43 aggregates propagation between cells. Immunofluorescence staining showed flotillin-1 colocalizes with mislocated TDP-43 in cytoplasm of cells in ALS-FTD-CSF group at day 21 (Figure 3A). Because protein aggregates transfer via exosomes happened in per-end stage and undergone cumbersome process, another possibility is that TDP-43 aggregates formed within one cell might access to the cytoplasm of neighboring uninfected cells by hijacking TNTs, as it was previously shown for prions [22] and Htt aggregates [23]. To demonstrate this possibility, U251 cells were cultured for 0, 1, 3, 5, 7, 10, 14, 18, 21, and 28 d, respectively, on petri dishes ready for imaging the formation of TNTs. By fluorescence microscopy, we visualized the expression of TNTs-like structure in ALS-FTD-CSF group starting to increase at day 3 (a relative percentage 117%), and reaching the peak at day 5 (a relative percentage 128%), then gradually decreasing from day 10 (a relative percentage 116%), at day 21 a relative percentage was still 116% (Figure 4A, 4B). In NC-CSF group the relative percentage of cells with TNTs-like structure kept unchanged during the incubation period (Figure 4B). These results indicate that ALS-FTD-CSF induced the generation of TNTs-like structure at early stage of incubation. We next determined whether TDP-43 aggregates transferred via TNTs-like structure. Immunofluorescence staining showed phalloidin-Alexa Fluor 488 colocalized with TDP-43 in the CSF-cultured cells, and TDP-43 aggregates were inside in TNTs-like structure (Figure 4C). Interestingly, the data showed that generation of TNTs-like structure occurs prior to the formation of TDP-43 aggregates. The TNTs-like structure expression kept the high level until TDP-43 aggregates could be detected, supporting that TNT-mediated transfer of TDP-43 proteinopathy happens at early stage.

### Incubation of ALS-FTD-CSF induces apoptosis, autophagy, and increases expression of TDP-43 CTFs in U251 cells

**Figure 5:**
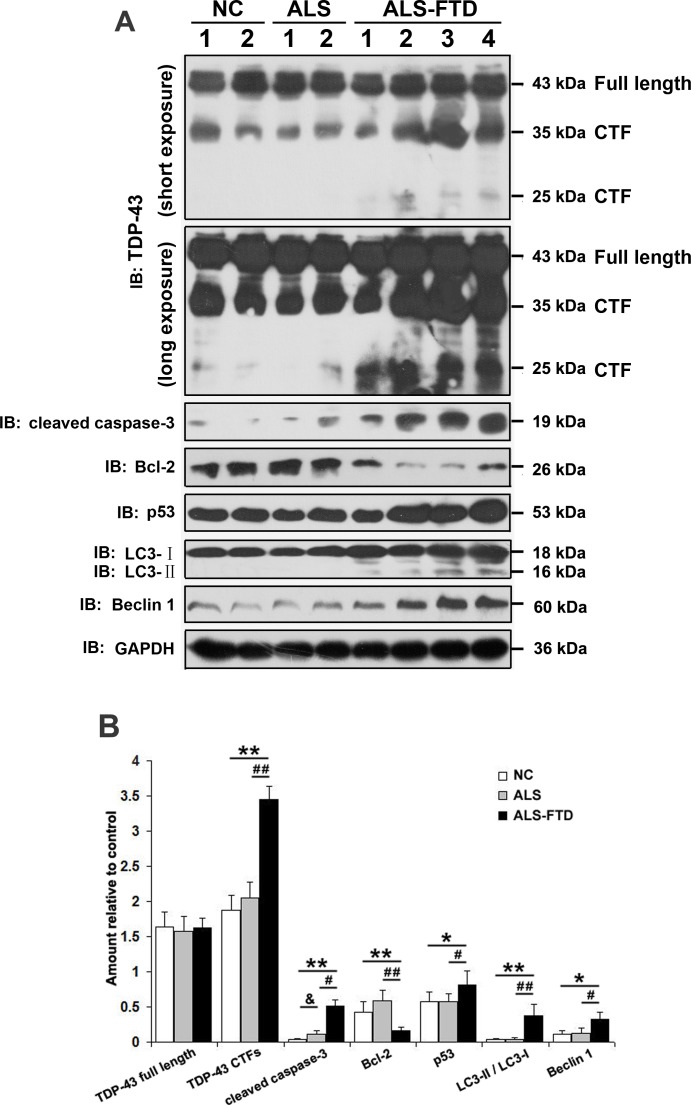
Exposure to ALS-FTD-CSF actives apoptosis and autophagy, and increases the expression of TDP-43 CTFs in U251 cells (A) Western blotting was performed using lysates of U251 cells following CSF incubation for 21 days. Cell lysates were examined by immunoblotting with the indicated antibodies. (B) Quantification showing significant increase in TDP-43 CTFs, cleaved caspase-3, p53, LC3II/LC3I, Beclin-1 levels in ALS-FTD-CSF-cultured cells compared to NC-CSF, whereas expression level of Bcl-2 was significantly decreased. Compared ALS-FTD-CSF group with ALS-CSF group, expression levels of TDP-43 CTFs, cleaved caspase-3, p53, LC3II/LC3I, Beclin-1 show significant increase, whereas Bcl-2 level decreases. Values shown are the mean ± SD from three experiments. Level of statistical significance, compared NC-CSF with ALS-CSF: ^&^ *p*< 0.05, ^&&^ *p*< 0.01; compared NC-CSF with ALS-FTD-CSF: *p< 0.05, **p< 0.01; compared ALS-CSF with ALS-FTD-CSF: ^#^ *p*< 0.05, ^##^ *p*< 0.01.

Intracellular aggregates are considered toxic to cells as they inducing apoptosis, autophagy, and other disturbances in internal environment [24]. In order to determine the apoptotic pathway induced by incubation of ALS-CSF and ALS-FTD-CSF in U251 cells, activation of caspase-3 along with Bcl-2 and p53 were assessed by Western blotting. As shown in Figure 5A, the expression levels of cleaved caspase-3 and p53 were increased, while the expression level of Bcl-2 decreased in ALS-FTD-CSF group, indicating that there was induction of apoptosis. In the ALS-CSF group, only the change of cleaved caspase-3 was shown significantly instead of the expression of other molecules examined (Figure 5A, 5B). We next assessed the expression of Beclin-1, an important autophagy effector that plays a key role in autophagosome formation, and LC3-II, which binds to the autophagosome membrane. We observed that the increased LC3-II expression (Figure 5A, 5B) resulting from incubation of ALS-FTD-CSF occurred concomitantly with an increase of Beclin-1 expression in U251 cells (Figure 5A, 5B). But, in ALS-CSF group, there is no significant change in the levels of autophagy associated protein (Figure 5B). These findings indicate that the two events, apoptosis and autophagy, co-exist in the ALS-FTD-CSF-cultured cells.

**Figure 6:**
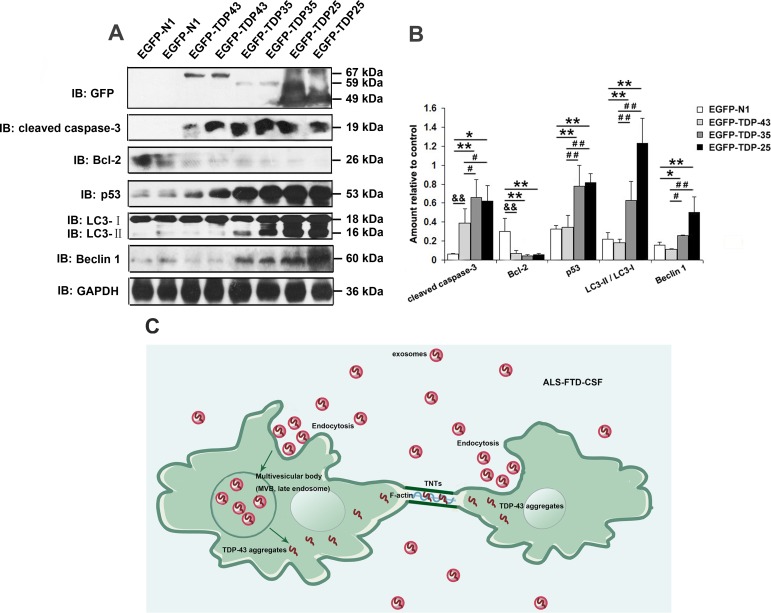
Overexpression of TDP-43 CTFs induces apoptosis and autophagy (A) Western blotting was performed using lysates of 293A cells following transfection of pEGFP, pEGFP-TDP-43, pEGFP-TDP-35, pEGFP-TDP-25 for 72 h. Each sample was probed with the indicated antibodies. (B) Quantification showing significant increase in cleaved caspase-3, p53, LC3II/LC3I, Beclin-1 levels in pEGFP-TDP35-transfected cells and pEGFP-TDP-25-transfected cells in comparison to pEGFP-transfected cells, whereas expression level of Bcl-2 was significantly decreased. Compared pEGFP-TDP-35-transfected cells or pEGFP-TDP-25-transfected cells with pEGFP-TDP-43-transfected cells, expression levels of cleaved caspase-3, p53, LC3II/LC3I, Beclin-1 significantly increase, whereas Bcl-2 level decrease. Values shown are the mean ± SD from three experiments. Level of statistical significance, compared pEGFP-transfected cells with pEGFP-TDP-35-transfected cells or pEGFP-TDP-25-transfected cells: *p< 0.05, **p< 0.01; compared pEGFP-transfected cells with pEGFP-TDP-43-transfected: ^&^ *p*< 0.05, ^&&^ *p*< 0.01; compared pEGFP-TDP-43-transfected cells with pEGFP-TDP-35-transfected cells or pEGFP-TDP-25-transfected cells: ^#^ *p*< 0.05, ^##^ *p*< 0.01. (C) TDP-43 aggregates spread among glioblastoma cells through exosomes and TNTs-like structure.

Our above-mentioned results showed that TDP-43 CTFs expression levels were increased in the exosomes derived from ALS-FTD-CSF, and earlier studies from others [25-27] demanstrated that TDP-43 CTFs were prone to form cytoplasmic aggregates. Then we detected the expression levels of TDP-43 CTFs in ALS-FTD-CSF-cultured U251 cells (Figure 5A). TDP-43 CTFs expression levels were increased in U251 cells after incubation of ALS-FTD-CSF for 21 days (Figure 5A, 5B). To further understand the effect of TDP-43 CTFs on cells, we next determined whether overexpression of full-length of TDP-43 or TDP-43 CTFs induced apoptosis and autophagy in 293A cells. As shown in Figure 6A and 6B, overexpression of GFP-TDP-43 induced an increase of cleaved caspase-3 and decrease of Bcl-2 expression level. But protein levels of p53, Beclin-1 and LC3 were unaffected compared with the GFP overexpressed cells. Immunoblotting analysis showed that expression levels of cleaved caspase-3, p53, LC3-II and Beclin-1 were increased, whereas expression level of Bcl-2 was decreased by overexpression of TDP-43 CTFs compared with GFP overexpressed cells (Figure 6B). Furthermore, TDP-43 CTFs overexpression induced the increase of cleaved caspase-3, p53, LC3-II and Beclin-1 compared with GFP-TDP-43 (Figure 6A, 6B). Based on these results, it is likely that TDP-43 CTFs could induce apoptosis and autophagy through a p53 dependent pathway.

## DISCUSSION

TDP-43 proteinopathies represent a novel class of neurodegenerative disorders akin to α-synucleinopathies and tauopathies. A broad clinic-pathological spectrum of a single disorder shares similar disease mechanisms linked to TDP-43 proteinopathies, such as ALS and FTD [28, 29]. Notwithstanding this view, the definite pathogenetic role of TDP-43 inclusions in neurodegenerative diseases is not yet established. Increased evidence indicates that gradual propagation of TDP-43 aggregates in a ‘prion-like propagation’ manner is a pathological process of sporadic ALS and FTD [21]. However, from the previous studies, an important difference between prion diseases and TDP-43 proteinopathies is that prions behave like infectious agents. Prions can be transmitted between individuals and even across species, and from the point of infection, often blood, CSF or a peripheral tissue, to CNS. Until now there is no direct evidence for transmitting of ALS or FTD between individuals. Using ALS-CSF in neuronal cultures has also been reported by various research groups; however, the research failed to detect the TDP-43 aggregates in the ALS-CSF-cultured cells [30-34]. Less resistant to endogenous proteases and other routes of pathogen elimination of misfolded protein are thought to be primary causes. In the current study, we have found that ALS-FTD-CSF served as a vehicle for the transmission of TDP-43 proteinopathies in U251 cells, but not ALS-CSF. Our data provide compelling evidence that ALS-FTD-CSF contains the transmissible ‘seed’, inducing seed-dependent aggregation of TDP-43 in astrocytes. Compared to the previous studies [30-34], we used CSF from patients concurrently suffered from ALS and FTD as culture medium, and extended the culture time to 28 days. These conditions make the possibility of ‘seed’ spreading from CSF to cultured cells. Then, we investigated what the ‘seed’ in the ALS-FTD-CSF is. Our results indicate that full-length TDP-43 and TDP-43 CTFs are enriched in exosomes isolated from ALS-FTD-CSF. TDP-43 was identified as the major component of the predominately cytoplasmic inclusions observed in the brain tissue of ALS and FTLD-U [6]. Moreover, TDP-43 CTFs are more prone to form the toxic, insoluble, and ubiquitin- and phospho-positive cytoplasmic inclusions within cells [18, 27, 35]. These findings suggest that full-length TDP-43 and TDP-43 CTFs in the exosomes from ALS-FTD-CSF act as a ‘seed’ which induces the prion-like aggregates of TDP-43 in the cultured U251 cells. However, there might be other potential factors act as “seed”, such as hyperphosphorylated full length TDP-43. Similarly, a recent study suggested insoluble TDP-43 from ALS or FTD brains could act as ‘seed’ to induce the generation of TDP-43 aggregates in SH-SY5Y cells overexpressing TDP-43 [10]. Kasai et al. reported increased TDP-43 protein in CSF of patients with ALS [36]. These results strongly support the idea that extracellular fluid from patients with TDP-43 proteinopathies contains the ‘seed’ for inducing cell-to-cell propagation of TDP-43 aggregates between neighbouring cells. Furthermore, we showed that incubation of ALS-FTD-CSF induced increase of exosomes containing cytoplasmic mislocalized TDP-43 in U251 cells. Taken together, our findings provide indirect evidence that exosomes mediate the spread of TDP-43 aggregates in ALS-FTD-CSF-cultured U251 cells. Therefore, interrupting transmission of exosomes containing pathological TDP-43 may become promising therapeutic strategies for inhibiting the progress of ALS and FTD.

According to the clinical symptoms, the spread mechanism of ALS and FTD lesion is explained by propagation, and classified as contiguous type and non-contiguous type [9]. It is surmised that the non-contiguous type propagation includes remote transfer of the toxic molecule through blood and CSF, and above findings demonstrated this hypothesis. TNTs and associated structures are new recognized ways for cell-to-cell communication [37]. They are F-actin-containing thin protrusions of the plasma membrane of a cell and support a direct physical connection between neighboring cells in local neuron pool [38, 39]. TNTs and associated structures serve as mediators for intercellular transfer of organelles as well as cytoplasmic molecules and misfolded proteins [15]. Previous research demonstrated that prions, amyloid-β, and polyglutamine aggregates were shown to transfer via TNTs between neuronal cells [15, 22, 23, 39]. Our results suggest that the generation of TNTs-like structures and exosomes is all promoted by ALS-FTD-CSF culture medium. TNTs-like structure and exosomes concurrently mediate the transfer of TDP-43 aggregates between ALS-FTD-CSF-cultured U251 cells. The generation of TNTs-like structure induced at onset stage of culture (5 days), and the expression of the TNTs-like structure maintains a high level until the formation of TDP-43 aggregates. In addition, the generation of exosomes is induced at a later stage of CSF culture (21 days), at the same time as the formation of TDP-43 aggregates. Our results suggest that exosomes and TNTs-like structure are two important mediators for the propagation of misfolded protein aggregates through CNS, and TNTs-like structure may predominantly mediate the spread of TDP-43 aggregates between neighboring cells in the early stage of disease progression.

ALS and FTD are characterized by gliosis and accumulation of large numbers of activated astrocytes, which typically surround the affected upper and lower motor neurons in ALS and neurons in the frontal and parietal lobes of FTD patients [40-42]. Reactive astrocytes in ALS and FTD contain cytoplasmic inclusions, the evidence sustaining an active role for astrocytes in the induction and propagation of misfolded protein aggregates in ALS and FTD [43-51]. We used human astrocytoma cells U251 to investigate the role of astrocytes in propagation of TDP-43 aggregates between cells. Our findings demonstrate that U251 cells were vulnerable to the incubation of ALS-FTD-CSF *in vitro*, characterized by decreased growth of cell, cytoskeletal abnormal and transmission of TDP-43 aggregates from ALS-FTD-CSF to the cultured cells. Previous research revealed that activated astrocytes become capable of damaging healthy neighboring motor neurons, and astrocytes start producing a host of toxic molecules [52], which in turn mediate the glial harmful action on neighboring neurons. Collectively, astrocytes may greatly contribute to drive the progression in TDP-43 proteinopathies via transferring TDP-43 aggregates from astrocytes to neurons, and further deciphering the interactions between motor neurons and astrocytes in neurodegenerative disease may reveal the basis for the progressive pathogenesis of the disease.

Autophagy and apoptosis are basic physiologic processes contributing to the maintenance of cellular homeostasis [53]. ALS and FTD have been considered as multi-factorial diseases with autophagy and apoptosis being proposed as some of its pathogenic reasons [53]. It has been established that elevated levels of aggregated proteins can cause compensatory activation of autophagy [54], and induction of autophagy have been shown in spinal neurons from animal models of ALS [55] and in post mortem samples from ALS cases [56]. The accumulation of aggregated misfolded proteins cause mitochondrial malfunction [57], which has been offered as a converging point for the multiple pathways that cause apoptosis activation and neuronal loss in ALS [58]. We report that apoptosis proteins, such as caspase-3, p53, Bcl-2, and macroautophagy-related proteins, such as LC3 and Beclin-1, are significantly regulated in ALS-CSF- and ALS-FTD-CSF-cultured U251 cells. Our findings indicate that activation of apoptosis and induction of macroautophagy were involved in the self-protective mechanism in neurodegenerative process of ALS-CSF- and ALS-FTD-CSF-cultured U251 cells.

## CONCLUSIONS

In conclusion, our results demonstrate that ALS-FTD-CSF containing the exosomes which display enrichment of full-length TDP-43 and TDP-43 CTFs induced the generation of TDP-43 aggregates in the cultured U251 cells. We further reveal that TNTs-like structure and exosomes mediated the spread of TDP-43 aggregates between ALS-FTD-CSF-cultured U251 cells. Moreover, our findings indicate that activation of apoptosis and induction of macroautophagy were involved in the pathological process of the ALS-CSF- and ALS-FTD-CSF-cultured cells. Thus, our data proffer the direct evidence for the ‘prion-like propagation’ mechanism of TDP-43 proteinopathies. Our work suggests that the understanding of the transfer mechanism of TDP-43 aggregates may provide the therapeutic strategy for patients with ALS and FTD.

## MATERIALS AND METHODS

### Patients and CSF collection

The healthy individuals and patients cohort in this study included 41 cases of Chinese descent. Clinical diagnoses were made by board certified neurologists according to consensus criteria for each disease. None of the cases was associated with any family history of ALS and FTD (genes tested via next generation sequencing: *SOD1, TARDBP, FUS, MAPT, GRN, CHMP2B, PSEN1, PSEN2, VCP, C9ORF72*), or clinical features suggestive of complications arising from other neurodegenerative diseases, including Parkinson's disease (PD), Alzheimer's disease (AD), multiple system atrophy (MSA), and progressive supranuclear palsy (PSP). All patients received a diagnosis of definite sporadic ALS according to the El Escorial criteria [11]. The ALS group consisted of 18 patients (11 men and 7 women). The mean (SD) age was 47 (6.2) years. Fifteen of 18 patients with ALS were classified as having spinal disease, three patients as having bulbar disease. The group of patients with ALS plus FTD comprised 8 patients (6 men and 2 women). The mean (SD) age of the patients was 66 (8.4, the same below) years. Eight patients were all classified as having the spinal form of ALS. These patients concurrently fulfilled diagnostic criteria for FTD [12]. Control subjects (10 men and 5 women) were 54 (4.2) years of age, without significant neurological symptoms, neurodegenerative or inflammatory diseases, and had normal results of a neurological examination.

CSF samples (between 10 and 15 ml per subject) were obtained by lumbar puncture. Only CSF samples without visible blood were used. All samples were aliquoted in small volumes and snap frozen in liquid nitrogen and stored at −80°C. All CSF samples used for the study were tested in duplicate. A retrospective analysis of the cases was completed at the end of the study.

These studies were approved by the ethical standards committee on human experimentation of Zhengzhou University. Written informed consent for research was obtained from all patients and healthy subjects participating in this study.

### CSF exposure and preparation of exosome fractions

Human glioma cell line U251 (ATCC, Rockville, MD) was routinely maintained in standard media, Dulbecco's modified Eagle's medium (DMEM) supplemented with 10% FBS (GIBCO, Invitrogen Corporation, Carlsbad, CA) at 37°C in 5% CO_2_ in a biosafety Level 2 containment. For CSF exposure, U251 cells were seeded into multi-well plates containing cover slips and allowed to grow until confluent (3 days), and then cells were exposed to 30% v/v (CSF/media) [13] of NC-CSF, ALS-CSF or ALS-FTD-CSF. On designated days (0, 1, 3, 5, 7, 10, 14, 18, 21, and 28), cover slips were removed from each treated group, and cells were prepared for assay. The groups in the study included normal control (NC: cells exposed to CSF of normal samples), ALS-CSF (ALS group: cells exposed to CSF of ALS samples), and ALS-FTD-CSF (ALS-FTD group: cells exposed to ALS-FTD-CSF samples).

Exosomes were prepared as described previously [14]. After thawing on ice, CSF was centrifuged at 15,000 × *g* for 10 min to pellet any shed cells, large membrane fragments, and other debris. The supernatant was then centrifuged at 200,000 × *g* for 60 min. The pellet was resuspended in 1×PBS and then re-centrifuged at 200,000 × *g* for 60 min before final resuspension in 1×PBS. The protein content of exosomes was determined using the BCA protein assay kit (Pierce, Rockford, IL, USA). Finally, the exosome fractions were dissolved in SDS sample buffer for immunoblotting.

### Immunofluorescence microscopy

After fixation for 15 min at room temperature, cells were treated with 0.25% Triton X-100 for 15 min and blocked by 4% FBS for 20 min, then incubated overnight at 4°C with the primary antibody following by incubation with secondary antibodies. Nuclei were stained with Hoechst 33258 dye (Calbiochem, San Diego, CA) at the concentration of 1 μg/ml for 5 min. To stain for TDP-43, the fixed cells were incubated with a rabbit polyclonal antibody (1:100; ProteinTech Group, Inc, Chicago, IL) in the blocking buffer at 4°C overnight. After overnight incubation, cells were washed and incubated with rhodamine-conjugated donkey anti-rabbit IgG (1:200; Santa Cruz Biotechnology, Santa Cruz, CA) at room temperature for 1 h. To stain flotillin-1, a mouse monoclonal anti-flotillin-1 antibody (1:50; Santa Cruz Biotechnology, Santa Cruz, CA) were used, and then cells were incubated with FITC-conjugated goat anti-mouse IgG (1:200; Santa Cruz Biotechnology, Santa Cruz, CA). The cells were visualized and photographed using a Nikon Labphoto-2 fluorescence microscope. The Image-Pro Plus 7.0 image analysis software was used to determine the ratio of TDP-43 fluorescence located in nucleus to cytoplasm in cells and the cell counting.

TNTs-like structure was fluorescently labeled with 1 μg/ml Wheat germ agglutinin (WGA) (Molecular Probes, Eugene, OR, USA) – Alexa Fluor 488 for 10 min at 37°C in the dark. F-actin was labeled with Alexa Fluor 488 phalloidin (1:100; Invitrogen AG, Basel, Switzerland). Cells were fixed with 4% PFA 10 min at room temperature, then incubated in 1×PBS with 0.1% Triton X-100 for 15 min and blocked in 10% bovine serum albumin for 1 h, and then cells were incubated with Alexa Fluor 488 phalloidin at room temperature for 1 h.

### Immunoblotting

Cells were collected from the plates and centrifugation at 500 × *g* for 5 min to sediment cells. The pellets were resuspended in TSPI buffer [50 mM Tris–;HCl (pH7.5), 150 mM NaCl, 1 mM EDTA, 1 mg/ml aprotinin, 10 mg/ml leupeptin, 0.5 mM Pefabloc SC, 10 mg/ml pepstatin, 1% NP-40]. After clearing the lysate by centrifugation, samples were heated in loading buffer, and equal amounts of protein were loaded and separated by SDS-PAGE. After transfer to nitrocellulose membranes, blots were blocked with 10% nonfat dry milk in TBST (0.25% Triton X-100 in PBS, pH 7.4) for 30 min, and then incubated with primary antibodies overnight at 4°C. After washing 3 times in TBST, the membrane was incubated with anti-rabbit IgG (1:5000; Cell Signaling, Beverly, MA) or anti-mouse IgG (1:5000; Cell Signaling, Beverly, MA) for 1 h. Membranes were washed three times and proteins were visualized after ECL (Pierce Chemical, Rockford, IL) treatment. The primary antibodies used were rabbit polyclonal anti-TDP-43 antibody (1:300; ProteinTech Group, Inc, Chicago, IL), rabbit polyclonal anti-flotillin-1 antibody (1:300; Abcam, Cambridge, UK ), mouse monoclonal anti-GAPDH antibody (1:2000; Cell Signaling, Beverly, MA), rabbit polyclonal anti-caspase-3 antibody (1:1000; Cell Signaling, Beverly, MA), mouse monoclonal anti-p53 antibody (1:500; Abcam, Cambridge, UK ), rabbit polyclonal anti-Bcl-2 antibody (1:500; Abcam, Cambridge, UK ), rabbit polyclonal anti-LC3 antibody (1:500; Cell Signaling, Beverly, MA), mouse monoclonal anti-Beclin-1 antibody (1:800; Cell Signaling, Beverly, MA) and rabbit polyclonal anti-GFP antibody (1:1000; Santa Cruz Biotechnology, Santa Cruz, CA).

### Statistical analysis

All statistical analyses were performed using SPSS statistical software package (SPSS version 8.0; SPSS Inc, Cary, NC). Data were shown as mean±SD. Multiple comparisons were tested with ANOVA and Bonferroni procedure. All tests were considered significant at *p-*value lower than 0.05.
